# Supplementary material for: Interactions of construal levels on programming ability and learning satisfaction: A case study of an Arduino course for junior high school students
Source: PLoS One. 2020 Aug 13;15(8):e0236500. doi: 10.1371/journal.pone.0236500 (PMC7425893; doi:10.1371/journal.pone.0236500)
Supplement: S1 Data — (PDF) [file pone.0236500.s001.pdf]

grade	name	CLT1	CLT2	CLT3	PRO	SAT1	SAT2	SAT3	SAT4	SAT5	SAT6	SAT7	SAT8	SAT9	SAT10	SAT11
8	A001	4	2	4	60	4	5	4	4	4	4	4	3	4	4	4
7	A002	4	4	5	70	4	5	4	5	5	5	4	5	4	5	5
8	A003	3	4	4	70	4	5	4	5	5	5	5	3	3	5	5
7	A004	5	1	5	90	3	4	5	5	5	4	4	4	5	4	4
8	A005	4	3	4	40	4	4	2	3	3	4	4	2	3	4	5
8	A006	4	5	5	60	4	5	4	4	4	5	5	5	3	5	5
7	A007	5	5	4	40	4	5	4	4	5	1	5	5	5	5	5
8	A008	4	4	4	80	4	4	5	4	4	5	4	3	2	4	4
8	A009	5	5	5	60	5	5	5	5	5	5	5	5	5	5	5
8	A010	5	5	5	70	5	5	3	5	5	5	5	5	5	5	5
8	A011	3	4	4	80	4	4	4	4	4	4	4	4	4	4	4
8	A012	4	2	4	40	4	5	4	4	5	5	4	4	5	4	4
7	A013	2	4	4	80	5	5	5	5	5	5	5	5	5	5	5
7	A014	3	3	3	80	5	5	5	5	5	5	5	5	5	5	5
8	A015	4	4	4	30	5	5	5	5	5	5	5	5	5	5	5
8	A016	4	4	4	80	4	4	5	5	4	4	5	4	5	4	4
8	A017	3	5	5	80	4	4	5	5	4	4	5	4	5	4	4
7	A018	3	3	4	80	3	4	4	4	4	4	4	4	4	4	3
8	A019	5	4	5	20	5	5	5	5	5	5	5	4	5	5	5
8	A020	5	5	5	100	5	5	5	5	5	5	5	5	5	5	5
8	A021	4	4	4	80	3	2	2	4	3	4	4	3	3	2	2
8	A022	4	4	4	80	3	2	2	4	3	4	4	3	3	2	2
7	A023	4	4	5	20	5	5	5	5	5	5	5	5	5	5	5
7	A024	3	5	5	60	5	5	4	4	5	4	5	4	4	5	5
8	A025	5	5	4	30	5	5	5	5	5	5	5	5	5	5	5
8	A026	4	4	4	90	5	4	4	4	4	5	5	5	5	5	5
8	A027	5	4	5	50	5	5	5	5	5	5	5	4	4	5	5
8	A028	4	5	5	100	5	5	5	5	5	5	5	2	4	5	5
7	A029	4	1	2	50	5	5	4	5	5	5	5	4	4	5	5
7	A030	4	5	4	40	5	4	4	4	5	5	5	5	2	5	5
8	A031	3	4	4	70	4	4	3	4	3	4	4	3	5	4	4
8	A032	3	4	4	60	2	3	4	4	2	4	2	3	4	4	2
7	A033	5	5	5	30	5	5	5	5	5	5	5	5	5	5	5
8	A034	4	4	4	80	4	4	3	4	5	4	3	4	4	4	4
8	A035	3	4	4	80	5	5	4	5	5	5	5	5	5	5	5

8	A036	3	4	4	40	4	4	4	4	4	4	4	4	5	4	5
7	A037	5	5	5	50	5	5	5	5	5	5	5	5	5	5	5
8	A038	4	3	4	30	2	3	4	2	3	4	4	2	2	4	4
8	A039	4	4	4	90	5	5	5	5	5	5	5	5	5	5	5
8	A040	5	4	5	80	5	5	4	4	5	5	5	4	5	5	5
8	A041	5	2	5	70	4	5	4	4	5	5	5	4	5	4	5
8	A042	3	2	4	60	3	3	3	3	4	4	4	4	3	4	4
8	A043	5	4	2	40	4	5	3	4	4	4	4	3	2	4	4
8	A044	5	5	5	70	5	5	5	5	5	5	5	5	5	5	5
7	A045	4	5	5	20	5	5	5	5	5	5	5	5	5	5	5
8	A046	5	4	4	60	5	5	5	5	5	5	5	5	5	5	5
7	A047	5	5	5	40	5	5	5	5	5	5	5	5	5	5	5
8	A048	4	5	5	70	4	5	4	4	5	5	5	4	4	5	5
8	A049	2	4	4	80	2	5	4	5	5	2	4	3	5	5	5
8	A050	4	2	4	70	4	4	4	5	4	4	4	3	4	4	4
8	A051	4	4	4	60	2	4	2	4	4	4	4	3	3	4	4
7	A052	5	4	4	70	4	5	4	4	4	4	4	4	4	4	4
7	A053	5	5	5	90	4	5	4	5	5	5	5	4	5	5	5
8	A054	3	3	3	40	3	5	1	4	5	5	4	3	5	4	5
8	A055	3	4	5	50	2	4	2	5	3	4	2	3	3	2	2
8	A056	4	4	4	70	4	4	5	5	4	4	4	3	5	4	4
8	A057	5	4	5	70	2	5	3	5	5	5	5	5	5	4	5
7	A058	4	5	5	60	4	5	3	5	4	5	5	4	4	5	5
8	A059	4	1	3	30	3	5	3	5	1	4	4	3	2	4	4
8	A060	5	5	5	40	4	5	1	4	5	5	5	4	5	5	5
7	A061	1	1	1	40	1	4	2	2	2	4	5	3	1	4	5
8	A062	5	4	5	80	4	4	3	5	4	4	4	3	4	4	4
8	A063	4	4	4	50	4	4	4	4	4	4	4	4	4	4	4
8	A064	4	2	3	80	3	4	3	5	5	4	4	4	4	4	4
8	A065	4	5	5	40	2	5	2	4	5	5	5	3	4	5	5
7	A066	5	5	5	60	5	5	4	5	5	5	5	5	5	5	5
8	A067	3	4	5	40	3	4	3	5	5	4	4	3	4	4	4
8	A068	3	2	4	50	4	4	4	4	4	5	5	3	4	4	5
8	A069	4	5	4	30	4	5	5	5	5	4	4	4	5	4	5
8	A070	4	3	3	10	3	4	3	4	3	4	2	3	3	3	3
8	A071	4	3	4	70	3	4	4	5	4	4	4	4	4	4	5

8	A072	3	3	4	60	2	2	2	4	3	3	2	4	3	3	2
8	A073	4	4	4	40	2	4	4	3	4	4	4	3	3	3	5
8	A074	4	3	3	60	4	4	4	5	5	5	5	5	5	4	5
8	A075	4	3	4	90	4	4	4	4	4	4	4	3	4	4	4
8	A076	3	3	3	40	1	4	1	4	3	4	4	3	4	4	3
8	A077	4	4	4	40	3	4	3	4	4	4	4	3	4	4	4
8	A078	2	2	5	40	4	4	4	3	4	4	5	5	4	3	5
8	A079	3	4	5	40	4	4	4	4	4	4	3	3	4	4	4
8	A080	5	1	5	100	4	5	5	5	5	5	5	3	1	5	5
8	A081	4	4	3	40	1	1	1	1	1	4	1	1	1	1	1
7	A082	4	2	4	60	4	4	4	4	4	4	4	4	4	4	4
8	A083	4	5	4	60	4	5	4	4	5	5	4	5	4	4	5
8	A084	4	4	4	100	5	5	5	4	5	5	4	2	5	5	5
8	A085	3	4	4	90	5	5	5	5	5	5	5	3	3	5	5
8	A086	4	5	4	100	5	5	5	5	5	5	5	5	5	5	5
8	A087	4	3	4	90	4	5	3	4	5	5	5	3	3	5	5
7	A088	4	5	5	30	5	5	5	5	5	5	5	5	5	5	5
7	A089	3	5	5	30	2	4	4	4	5	4	3	4	5	4	5
8	A090	4	4	3	20	4	4	4	4	4	4	4	4	4	4	4
8	A091	4	4	5	40	5	4	4	4	4	4	4	4	3	5	4
8	A092	2	4	5	40	5	5	5	5	5	5	5	5	5	5	5
7	A093	2	4	2	30	2	5	1	5	5	5	5	5	5	4	2
8	A094	3	4	4	60	3	4	4	5	4	4	3	4	4	4	4
7	A095	4	2	4	70	4	4	4	4	4	4	4	4	4	4	4
