# Supplementary material for: Interactions of construal levels on programming ability and learning satisfaction: A case study of an Arduino course for junior high school students
Source: PLoS One. 2020 Aug 13;15(8):e0236500. doi: 10.1371/journal.pone.0236500 (PMC7425893; doi:10.1371/journal.pone.0236500)
Supplement: Attachment — Submitted filename: renamed_1d834.docx [file pone.0236500.s002.docx]

**Reviewer #1**

**Generally, this paper is well-structured and has valid experimental foundations.**

Thank you for your positive assessment of our manuscript; we appreciate your time, effort, and insight in reviewing our manuscript, which has led to an improved paper. We respond to your suggestions below.

**1) The abstract has 183 words; it is relatively lengthy. The language in this session is not concise enough. Some important information that the audience expects to know cannot be found in the abstract, like the sampling number and the questionnaires. While some less significant information, for example, the features of Arduino, could be introduced in somewhere else to make the abstract more effective.**

Thank you for the excellent suggestion. We condensed the abstract from 183 to 168 words. Also, we highlighted the sampling number and the questionnaires, as suggested. Furthermore, we moved the features of Arduino to the literature review.

**2) Chinglish is a crucial issue in the introduction section. Using “the Education Bureau of Taiwan”, “for two technology courses weekly” et al., can make the language more readable.**

**3) In fact, some other language errors can be found throughout the manuscript, the review suggests the authors to carefully proofread the article.**

Indeed, there is much room for improvement in English writing; we changed some wordings to make the paper more readable. We also asked for help from native English speakers for professional proofreading.

**4) The references in the literature review part need to be strengthened. For example, there should be citations for the claim “Assessment of students’ LS from multiple perspectives (students’ LS concerning PLA, their schools, and their teachers) revealed…”.**

Thank you for this reminder; we tried our best to reorganize and strengthen the literature review part.

**5) Construal Level Theory has been introduced both in the introduction part and literature review part with considerable space, the reviewer suggests combining the two parts and highlighting the related studies.**

We reorganized the introduction of CLT in the literature review section and highlighted the related studies.

**6) It is confusing to have “Moore motivated” in Figure 1.**

We corrected this obvious typo.

**7) Please define the relationship of “Arduino” and “Scratch”, if we have “Arduino” both in the article title and abstract, we expect to have the literature review section named “Arduino” instead of “Scratch”. Or the authors may think another way to represent them, just keep consistent.**

We added a literature review section named “Scratch and Arduino” to introduce the relationship between them.

**8) The reviewer recommends moving the position of “Operational definitions of research constructs” section to the front to assist the audience to understand the concepts in a logical way.**

We followed this excellent suggestion.

**9) Please keep the table font consistent.**

We applied the template to make the font consistent.

**10) The “first, second, third” point sentences in the conclusion part could be improved.**

We rewrote the conclusion part.

**11) Need citations for the sentence “A series of studies revealed that individuals have higher expectations of long-distance performance than of short-distance performance”, and the claim of “cold feet”.**

We specified the viewpoint was sourced majorly from “Liberman, N., & Trope, Y., & Stephan, E. (2007).”

**12) This article could be more insightful if the authors integrate the computational thinking element in the discussion part.**

We supplemented this great suggestion in both the introduction in stage 3 of the research procedure and “theoretical implications.”

**13) It is suggestible to change the position of the last two paragraphs of the article and frame**

We rewrote the final section.

**Reviewer #2**

**This study tries to explore the effects of students’ construal levels and programming language ability on learning satisfaction. However, the current presentation and results of the manuscript may not live up to expectation.**

We appreciate this motivating comment. Thank you for your time, effort, and insight in reviewing our manuscript, which has led to an improved paper. We respond to your suggestions below.

**1. Research model and research hypotheses, that drive the paper, should be built from an ongoing and pertinent bibliography (up to 2020). Identifying a research gap is not enough; key is showing its significance to the field.**

We appreciate this great suggestion, and we added one reference paper published in 2020. In the abstract and “theoretical implications” section, we emphasized, “This study, therefore, explored the effects of students' construal level that not only fill the research gap but also open a new avenue for the improvements of programming ability.”

**2. The research process and methodology are not clearly presented and described, and current descriptions are quite puzzling. Several issues should be clearly stated. First, how do you measure programming language ability? What type of online test were used? Was programming language ability measured only before Arduino course? How about after taking the Arduino course? I say this cause I think that the relationship between the change in programming language ability and the learning satisfaction may be worth investigated.**

Indeed, the original version of the research process are not clearly presented and described. We rewrote the whole section to respond to your essential suggestions. Investigation of the relationship between programming ability and learning satisfaction, plus the moderating effect of construal levels, revealed insightful findings.

**3. In addition, it is not very clear how you measured construal levels, did you just used the three questions in Table 2? Are there any literature supporting your use of such questions as measures of construct levels? Have you also used any qualitative method? Also, as you mentioned, “According to the results, students were divided into high- and low- construal level group”, how did you defined high- and low- construal level?**

To measure students’ construal levels, we referenced Ho et al.’s (2015) approach. We carefully designed three questions. Besides, the instructor introduced a scenario describing two representative construal level situations. In this way, the measurement of the construal level concerns both quantitative and qualitative issues. We used the median score of construal level construct to divide students into high- and low- CL group.

**4. As the authors mentioned about a use of cooperative learning approach for course teaching, how did you divide different groups? More detailed information should be provided.**

We detailed this part on the research procedure, as “Stage 3 was the teaching of Arduino by the CL method [19,38,44], and the "Learning Together" model [18], where students are divided into heterogeneous groups of four or five randomly and given a study assignment sheet; each group must complete a common assignment.”

**5. There is something wrong with Table 4, which is expected to present SEM results, however, currently, it is the same with Table 3. Without complete statistical results, it is not possible to judge your findings. Please do carefully check your manuscript before submitting it.**

We are deeply sorry for this big mistake and corrected.

**6. Besides, as for the sample size, I would say that a sample of 95 for conducting SEM would not be very persuasive. At least you can justify that the use of a sample of 95 is enough to clarify your analysis.**

Since this is an experimental study, the sample size is limited. We described reasons to overcome this problem by adopting PLS-SEM instead of CB-SEM. We also listed this issue in the “Limitations and directions for future research” section.

**7. The presentation and structure as well as the language use should be further improved.**

Indeed, the presentation and structure, as well as language use, have much room for improvement. We tried our best to enhance the presentation and structure. We also asked for help from native English speakers for professional proofreading.
