# Supplementary material for: Interactions of construal levels on programming ability and learning satisfaction: A case study of an Arduino course for junior high school students
Source: PLoS One. 2020 Aug 13;15(8):e0236500. doi: 10.1371/journal.pone.0236500 (PMC7425893; doi:10.1371/journal.pone.0236500)
Supplement: Attachment — Submitted filename: Review paper Plos One.docx [file pone.0236500.s003.docx]

The manuscript has been improved a lot compared with the original version. I appreciate the efforts that the authors have made to make the article more publishable in the journal. However, still, there are some points that may need more attention from the authors.

1) The second sentence of the abstract makes the logic confusing. It is unrelated to the theme of the article, whether remove it or revise it.
2) The tense of the penultimate sentence of the abstract is inappropriate.
3) The third paragraph of the introduction section lacks a theme sentence at the beginning of the paragraph. In fact, the review can see only limited value of this paragraph, it is necessary for it to be there?
4) Similarly, add the theme sentences for the many paragraphs in the literature review section. Not just list different publications to compose the whole paragraph.
5) The hypothesized relationship structure indicates that this study is not investigating the “Effects of Construal Levels on Programming Ability and Learning Satisfaction”, but the “Interactions of Construal Levels on Programming Ability and Learning Satisfaction”, think about correcting the title to be consistent with the research itself.
6) Please change the size of table one to make it tidier, remove the shadow of table 2 and table 3. Address front size problems in the reference section. Not happy to see this kind of errors at this stage.

Good luck!
