# Supplementary material for: Interactions of construal levels on programming ability and learning satisfaction: A case study of an Arduino course for junior high school students
Source: PLoS One. 2020 Aug 13;15(8):e0236500. doi: 10.1371/journal.pone.0236500 (PMC7425893; doi:10.1371/journal.pone.0236500)
Supplement: Attachment — Submitted filename: renamed_b3eb6.docx [file pone.0236500.s004.docx]

**Reviewer #1**

**The manuscript has been improved a lot compared with the original version. I appreciate the efforts that the authors have made to make the article more publishable in the journal. However, still, there are some points that may need more attention from the authors.**

Thank you for your warm encouragement for our efforts. We appreciate your time, energy, and insight in reviewing our manuscript, which has led to an improved paper. We responded to your suggestions below.

**1) The second sentence of the abstract makes the logic confusing. It is unrelated to the theme of the article, whether remove it or revise it.**

We agree with your suggestion and removed the sentence.

**2) The tense of the penultimate sentence of the abstract is inappropriate.**

We corrected this mistake.

**3) The third paragraph of the introduction section lacks a theme sentence at the beginning of the paragraph. In fact, the review can see only limited value of this paragraph, it is necessary for it to be there?**

**4) Similarly, add the theme sentences for the many paragraphs in the literature review section. Not just list different publications to compose the whole paragraph.**

Indeed, there is much room for improvement in the writing of this paper; we changed some wordings and made theme sentences more significant to make the article more readable. We also asked for help from native English speakers for professional proofreading. Your care and suggestion for the novice researcher are very touching.

**5) The hypothesized relationship structure indicates that this study is not investigating the “Effects of Construal Levels on Programming Ability and Learning Satisfaction”, but the “Interactions of Construal Levels on Programming Ability and Learning Satisfaction,” think about correcting the title to be consistent with the research itself.**

Thank you for this excellent suggestion. We corrected the title to be consistent with the research itself.

**6) Please change the size of table one to make it tidier, remove the shadow of table 2 and table 3. Address front size problems in the reference section. Not happy to see this kind of errors at this stage.**

Thank you for this diligent remind, we tried our best to correct all errors.

**Reviewer #2**

**Most of the major concerns have been solved. There are still minor issues.**

Thank you for your warm support; we appreciate the great help from you. We responded to your suggestions below.

**First, some of the dated references in the papers could be replaced by more recent ones (including 2018-20).**

We replaced some references with more recent ones; please refer to

references from 2018-20 as listed in #6,8,25,37,39,41,46.

**Besides, presentation should be further improved. For example, please provide page number for: Sweeney and Ingram [39] defined LS as the “perception of enjoyment and accomplishment that learners develop in learning environments.”**

**The same for: Similarly, Kuo, Walker, Belland, Schroder, and Kuo [26] defined LS as “student perceptions of the extent to which their learning experiences were helpful and enjoyable.”**

Thank you for this industrious remind; we followed this suggestion.

The 39th reference has been changed to a newer one, and the content is on page 590.

The content of the 26th reference is on page 164.

**Also, inconsistent tense use issues, e.g., tense of “provide” and “encouraged” in the following paragraph should be consistent.**

**“Parker [35] indicated that the heterogeneous groups in cooperative learning provide an environment that enables students to learn alongside their peers, support one another, offer constructive criticism, share their views, and share their results. Johnson and Johnson [18] reported that cooperative learning encouraged face-to-face interaction to solve problems, offer mutual assistance, and share ideas.”**

**In addition, formation issue, e.g., “Figure 3 details” and “the stages of the experiment.” should be aligned.**

**Please double-check the whole manuscript.**

We double-checked the whole manuscript to correct all related issues and asked for help from native English speakers for professional proofreading.
